# Chronic α5‐GABA‐A Receptor Potentiation Promotes Mouse Adult Hippocampal Neurogenesis

**DOI:** 10.1002/hipo.70019

**Published:** 2025-06-13

**Authors:** Thomas D. Prevot, Michael Marcotte, Denis J. David, Indira Mendez‐David, Md Yeunus Mian, James M. Cook, Jean‐Philippe Guilloux, Etienne Sibille

**Affiliations:** ^1^ Campbell Family Mental Health Research Institute of CAMH Toronto Ontario Canada; ^2^ Department of Psychiatry University of Toronto Toronto Ontario Canada; ^3^ Department of Pharmacology and Toxicology University of Toronto Toronto Ontario Canada; ^4^ Faculté de Pharmacie Université Paris‐Saclay, UVSQ, Centre de Recherche en Epidémiologie et Santé Des Populations (CESP) Orsay France; ^5^ Department of Chemistry and Biochemistry University of Wisconsin– Milwaukee Wisconsin USA

**Keywords:** adult hippocampal neurogenesis, fluoxetine, GABA, hippocampus, preclinical, α5‐γ‐aminobutyric acid type a (GABAA) receptor

## Abstract

Several lines of evidence implicate adult hippocampal neurogenesis (AHN) in cognitive functions, in mood‐ and anxiety‐related behaviors, and in the therapeutic effects of antidepressants. Augmenting α5‐γ‐Aminobutyric acid type A (GABAA) receptor function has shown neurotrophic effects in stress and aged models, but its impact on mouse AHN remains unknown. Adult male 129S6/SvEvTac mice (*n* = 30 total) were treated for 6 weeks with GL‐II‐73, an α5‐GABAA‐R‐positive allosteric modulator (α5‐PAM) [30 mg/kg, per os, (P.O.)] or fluoxetine, a prototypical selective serotonin reuptake inhibitor known to increase AHN (18 mg/kg, P.O.). Proliferation in the subgranular zone of the dentate gyrus (DG) was assessed by the level of Ki67, a marker of dividing cells; survival of the young neurons was assessed by retention of the 5‐Bromo‐2´‐Deoxyuridine (BrdU) nucleotide analog injected 2 weeks before sacrifice. Finally, maturation of young adult‐born neurons was evaluated by measuring the fraction of BrdU‐positive cells that are also DCX and/or NeuN‐positive, capturing overall maturation and speed of maturation. Similarly to fluoxetine, a chronic treatment with GL‐II‐73 stimulated all stages of AHN, significantly increasing neuronal progenitor proliferation, survival of adult‐born granule cells, and maturation of young neurons in the DG of the hippocampus. Chronic treatment with GL‐II‐73, a α5‐GABAA‐R‐positive allosteric modulator, increased AHN, including cellular proliferation, survival, and maturation of newborn neurons, to levels comparable to fluoxetine.

1

Two regions continue to generate new neurons into old age in the adult mammalian brain: the subventricular zone (SVZ) and the subgranular zone (SGZ) of the hippocampus, responsible for the formation of adult‐born granule cells in the dentate gyrus (DG). In mice, both the SVZ and SGZ neural stem cell niches produce new neurons throughout life, although their numbers significantly decrease with age (reviewed by Snyder (2019)). In the human SGZ, most studies indicate that adult‐born granule cells are generated until old age (Boldrini et al. 2018; Flor‐García et al. 2020; Indira Mendez‐David et al. 2023; Mendez‐David et al. 2022; Tartt et al. 2018; Zhou et al. 2022).

The newly born cells migrate into the DG, express neuronal markers, receive synaptic inputs, extend axons along the mossy fibers tract, and exhibit electrophysiological properties similar to those of mature dentate granule neurons (Malberg et al. 2000; Santarelli et al. 2003). Therefore, AHN provides the brain with appropriate adaptability in structure and functions in response to stimuli (Lledo et al. 2006), including in mood regulation and cognitive functions. AHN is reduced in various brain disorders and conditions, including Alzheimer's disease, depression, schizophrenia, and overall stress‐related disorders and chronological aging (Choi and Tanzi 2023; Tartt et al. 2022). Frontline treatments for MDD, such as selective serotonin reuptake inhibitors (SSRIs) and other therapeutics with different mechanisms of action, increase AHN in the mouse brain (Guilloux et al. 2013; Guirado et al. 2012; Malberg et al. 2000; Mendez‐David et al. 2013; Rainer et al. 2012; Santarelli et al. 2003; Wang et al. 2008) and, potentially, in the adult human brain (Maura Boldrini et al. 2009; David et al. 2009; Indira Mendez‐David et al. 2023; Olivas‐Cano et al. 2023; Sachs and Caron 2014; Tartt et al. 2022). Moreover, blockade of AHN partially impairs the efficacy of SSRIs in mice, suggesting neurogenesis‐dependent mechanisms of antidepressant action (David et al. 2009; Malberg et al. 2000; Mendez‐David et al. 2017; Santarelli et al. 2003).

γ‐aminobutyric acid (GABA) levels and markers of GABAergic interneurons are also reduced in depression (Prévot and Sibille 2021). GABA regulates the initial transition from quiescence to neural differentiation and neuronal maturation process (Tozuka et al. 2005). GABA is initially excitatory in the developing brain on immature neurons (neuroblasts and neural stem cells) (Pontes et al. 2013). It becomes the main inhibitory neurotransmitter in mature neurons due to a switch in the expression of Na+/K+/2Cl − co‐transporter NKCC1, driving Cl − influx, and neuron‐specific K+/Cl − co‐transporter KCC2, driving Cl − efflux (Pfeffer et al. 2009). GABAergic excitation in immature neurons may facilitate AHN during early hippocampal development (Pfeffer et al. 2009). Its role in the adult hippocampus may thus depend on the maturation state of neurons.

GABA signals through ion channel GABAA receptors and metabotropic GABAB receptors. GABAA receptors contain α, β, γ, δ, ε, θ, π and ρ subunits assembled into pentamers (Mohler 2006). Among the GABAA receptor subtypes, the one expressing the α5‐subunit is highly prevalent in the hippocampus, including in the granule cell layer of the DG (Palpagama et al. 2019) where AHN occurs, and is expressed on neural stem cells, early progenitors in the SGZ (Pallotto and Deprez 2014) and on immature granule cells (Hochgerner et al. 2018). Novel compounds acting as positive allosteric modulators of the α5‐GABAA receptors (α5‐PAM) were recently developed. The α5‐PAM compound GL‐II‐73 was shown to have antidepressant and pro‐cognitive effects in mouse models (Bernardo et al. 2022; Prevot et al. 2019; Prevot et al. 2021). In addition, chronic administration of GL‐II‐73 reversed the loss of spine density and dendritic length on mature hippocampal neurons induced by age (Prevot et al. 2021), chronic stress (Bernardo et al. 2022) and Alzheimer's disease pathology (Bernardo et al. 2022) in mice. Considering the role of GABA on differentiation of neural stem cells via its excitatory effect on immature cells, the high expression of α5‐GABAA receptor in the hippocampus, and the neurotrophic effects of GL‐II‐73 in the cortex and hippocampus, we hypothesize that α5‐PAM may also promote DG AHN.

To test this hypothesis, we used a 129S6/SvEvTac mouse strain, a mouse strain with reported low AHN baseline (for review Mendez‐David et al. (2013)), treated with GL‐II‐73 or fluoxetine (as a positive control) for 6 weeks in the drinking water (Figure 1A) at 30 mg/kg as in (Prevot et al. 2021), and measured cell proliferation, cell survival, and maturation of newly born neurons in the DG of the hippocampus. Immunostaining against Ki‐67, a nuclear protein associated with cellular proliferation, revealed significant increases in the proliferation of newborn cells after chronic treatment with both fluoxetine and GL‐II‐73 (Figure 1B and Table S1; ANOVA: F_(2;23)_ = 12.21; *p* = 0.0002; effect of fluoxetine: *p* = 0.0011; effect of GL‐II‐73: *p* = 0.0001). There was no difference in Ki67 levels between the fluoxetine and GL‐II‐73‐treated groups (*p* > 0.05). Regional proliferation of neural progenitors in the dorsal and ventral segments of the DG was also analyzed (Figure S1A; Table S2). Two‐way ANOVA confirmed an effect of treatment (F_(2,23)_ = 10.67; *p* = 0.0005) and region (F_(1,23)_ = 38.69; *p* < 0.0001), though no significant interaction effect (F_(2,23)_ = 1.828; *p* = 0.1834). Post hoc analysis confirmed increased Ki67 labeling with fluoxetine and GL‐II‐73 treatments compared to vehicle. Similar results were obtained in both the dorsal and the ventral segments, with post hoc analyses showing higher Ki67 labeling in the ventral segment compared to the dorsal segment in both fluoxetine and GL‐II‐73‐treated mice.

**FIGURE 1:**
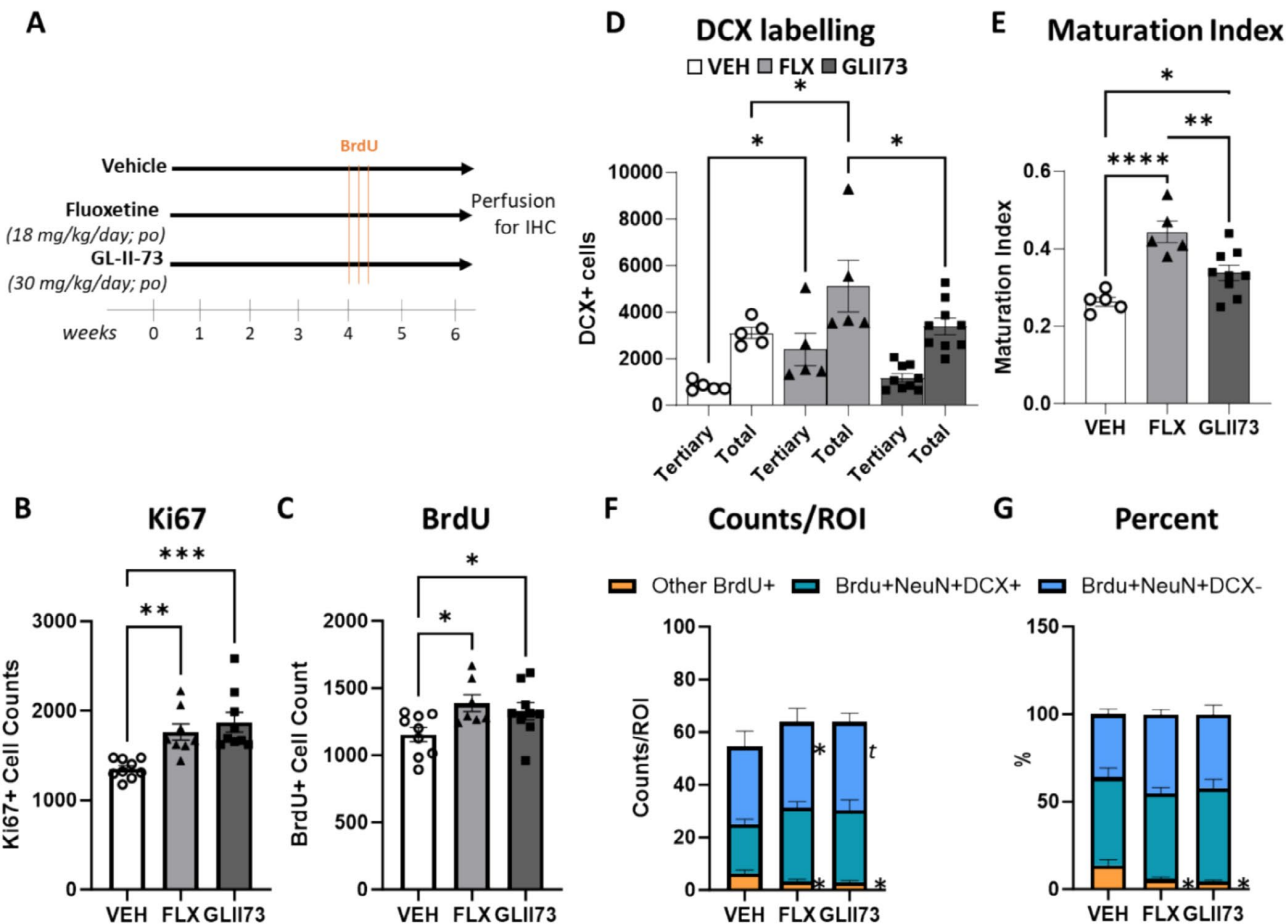
Effects of GL‐II‐73 on proliferation, survival, and maturation of newborn neurons in the mouse hippocampus, compared to fluoxetine. *The experimental design is present*ed in A. Two‐month‐old mice were treated P.O. with GL‐II‐73, fluoxetine, or vehicle for 6 weeks. At 4 weeks from commencement of treatment, mice were given 6 injections of BrdU (100 mg/kg) over 3 days. Mice were sacrificed 2 weeks later and perfused with PFA. Brains were collected for immunohistochemistry. Chronic treatment with GLII73 increased proliferation of newborn cells (Ki67+ labeling; B), survival of the young adult‐born granule cells (BrdU+ labeling; C) and maturation of young neurons (DCX+ labeling; D–E). Sections of mouse brains treated with fluoxetine, GL‐II‐73, or vehicle control were stained for BrdU, NeuN, and DCX. Quantification of BrdU+ cells per region of interest expressing DCX and NeuN revealed that fluoxetine and GL‐II‐73 decreased the number of other BrdU+. Mean ± SEM. *p < 0.05, **p < 0.01, ***p < 0.001 compared to vehicle.

The survival of the young adult‐born granule cells, evaluated following 5‐Bromo‐2‐Deoxyuridine (BrdU) administration twice daily at 100 mg/kg intraperitoneally, for three consecutive days, 2 weeks before killing, revealed a significant and similar increase in BrdU+ cells in mice treated with either fluoxetine or GL‐II‐73 (ANOVA: F_(2,22)_ = 3.99; *p* = 0.03; post hoc compared to vehicle: *p* = 0.014, and *p* = 0.048, respectively; Figure 1C and Table S3). Upon dividing the data into dorsal and ventral regions of the hippocampus (Figure S1B, and Table S4), 2‐way ANOVA confirmed a significant effect of treatment (F_(2,22)_ = 3.99; *p* = 0.03), and showed a significant effect of region (F_(1,22)_ = 180.3; *p* = < 0.0001) and a trend toward a treatment × region interaction (F_(2,22)_ = 3.35; *p* = 0.053). GL‐II‐73 increased the number of BrdU+ cells in the dorsal region (*p* = 0.033) but not in the ventral region (*p* = 0.32). Conversely, fluoxetine increased the number of proliferating precursors in the ventral region (*p* = 0.003) but showed no effect in the dorsal region.

Staining with antibodies targeting the microtubule associated protein doublecortin (DCX), a marker of immature neurons, revealed a significant effect of fluoxetine and GL‐II‐73 on neuronal maturation (Figures 1D andS2, and Table S5). DCX+ cells were subcategorized according to their morphology, including neurons with complex tertiary dendrites representing more mature neurons (Wang et al. 2008). A two‐way ANOVA on DCX+ cells revealed a significant effect of treatment (F_(2,32)_ = 6.527, *p* = 0.0042) and of cell morphology (F_(1,32)_ = 34.36, *p* < 0.0001) with no interaction (F_(2,32)_ = 0.1489, *p* = 0.86). Post hoc analysis revealed a significant increase of total DCX+ cells and DCX+ cells with tertiary dendrites after chronic fluoxetine (*p* = 0.0134 and *p* = 0.048, selectively) but not after chronic GL‐II‐73 (*p* = 0.25). A maturation index was derived by dividing the number of DCX positive cells with tertiary dendrites by the total number of DCX cells (Figure 1E; and Table S6). There was a significant effect of treatment on maturation index (F_(2,16)_ = 14.12, *p* = 0.0003) and post hoc testing showed that both fluoxetine (*p* < 0.0001) and GL‐II‐73 (*p* = 0.0261) increased the index. The data were further stratified along the dorsal ventral axis (Figure S3, and Table S7). A two‐way ANOVA revealed a significant effect of treatment (ANOVA: F_(2,15)_ = 13.85; *p* = 0.0004), region (ANOVA: F_(1,15)_ = 7.703; *p* = 0.014) but no interaction.

To further characterize the fate of dividing cells and their trajectory into mature neurons in the hippocampus of mice treated with GL‐II‐73 or fluoxetine, a triple labeling experiment was performed on a subset of tissue from the same mice (Figures 1F,G, S4, and Table S8–S11), with BrdU+ cells representing surviving cells, BrdU+NeuN+DCX+ maturing neurons, and BrdU+NeuN+DCX‐mature neurons. Analyses of the expression of BrdU+DCX‐NeuN+ confirmed a significant effect of cell maturation steps (ANOVA F_(2,20)_ = 3.79; *p* = 0.0401). Post hoc analyses showed a significant increase in mature neurons (BrdU+DCX‐NeuN+ cells) in the fluoxetine‐treated group (*p* = 0.014) and a trend‐level increase in the GL‐II‐73‐treated group (*p* = 0.068) compared to the vehicle group. In addition, there was a significant reduction of nonmature neurons (BrdU+DCX‐NeuN‐ cells) in the fluoxetine and GL‐II‐73 treatment groups (*ps* < 0.01). Analyses applied to the proportion of cells within each treatment group indirectly confirmed the effects of GL‐II‐73 and fluoxetine on maturation (ANOVA: F_(2,20)_ = 5.393; *p* = 0.0134), marked by a reduction in the proportion of nonmature neurons (other BrdU+ cells) in the fluoxetine (*p* = 0.0164) and GL‐II‐73 (*p* = 0.0061) treatment groups.

In summary, this study suggests that chronic treatment with GL‐II‐73, a novel small‐molecule positive allosteric modulator of the α5‐GABAA‐R, increases all AHN stages, including cellular proliferation of newborn cells (via increased Ki67 labeling), plausibly survival (via BrdU labeling), possibly linked to increased proliferation) and maturation of newborn neurons (via DCX labeling). The effect of GL‐II‐73 on neuronal maturation was significant but moderate compared to the robust effect of fluoxetine (confirming previous studies (Mendez‐David et al. 2023; Wang et al. 2008)). Although fluoxetine (Micheli et al. 2018; Olivas‐Cano et al. 2023; Sachs and Caron 2014) and other antidepressants, such as venlafaxine (Belovicova et al. 2017), vortioxetine (Bennabi et al. 2019; Felice et al. 2018), paroxetine (Jahromi et al. 2016) and other molecules with different mechanisms of action, such as metformin (J. Wang et al. 2012), trametinib (a kinase inhibitor for cancer) (Kim et al. 2023) and psychedelics like psilocybin (Catlow et al. 2013; Lino de Oliveira et al. 2020; Malberg 2004) increase AHN in the adult brain, little is known about GABAergic drugs contribution to AHN. GABA was shown to regulate AHN (Catavero et al. 2018; Drew et al. 2016). Endozepine diazepam‐binding‐inhibitor, acting as a γ2 negative allosteric modulator, limits the proliferation of neurons (Everlien et al. 2022), and diazepam, an α1/2/3/5 positive allosteric modulator, has no effect on AHN (Furukawa et al. 2021) and was reported in some instances to reduce it (Villasana et al. 2019). Hence, this is the first report showing that augmenting GABAergic function, in this case through α5‐GABAA receptor, increases AHN in the adult rodent hippocampus.

To better capture the trajectory of pharmacological effects of AHN steps, the triple labeling study showed that treatment with GL‐II‐73 and fluoxetine reduced the proportion of cells not expressing NeuN, that is, the cells that are in an early stage of maturation. Conversely, both treatments increased the proportion of cells being DCX‐/NeuN+, that is, the cells that reach the end of the maturation steps and become neurons. This suggests that treatment with either GL‐II‐73 or fluoxetine accelerates the maturation processes compared to vehicle treatment. While these findings confirm the previous evidence of the neurogenic effect of fluoxetine, they highlight for the first time that a small molecule facilitating GABAergic activity can accelerate cellular processes involved in AHN. However, it remains unclear whether the effect of GL‐II‐73 is caused by activity on progenitor cells or through other mechanisms that increase AHN (e.g., activity on neural stem cells). Investigating hippocampal single‐cell RNAseq data (Hochgerner et al. 2018) (Linnarssonlab.org) indicates that GABRA5, the gene coding for the α5‐subunit of GABAA‐Rs, is expressed in mature and immature granule cells and in immature pyramidal neurons (data not shown). Since GABA is likely to have an excitatory effect on these developing neurons (Kaila et al. 2014; Pontes et al. 2013), it is probable that the effects of GL‐II‐73 may be direct and through stimulatory activity mediated by α5‐GABAA‐Rs receptor on immature pyramidal and granule cells.

Importantly, studies have shown that the antidepressant action of fluoxetine is partially due to its impact on AHN, as blockade of AHN partially impairs the behavioral efficacy of SSRIs in mice (David et al. 2009; Malberg et al. 2000; Mendez‐David et al. 2017; Santarelli et al. 2003). Similar reasoning could be applied to GL‐II‐73. Indeed, GL‐II‐73 was shown to have procognitive effects and antidepressant potential (Prevot et al. 2019). Therefore, it would be interesting to investigate whether these behavioral effects are dependent on the AHN‐related effects of GL‐II‐73. Some evidence suggests that the effects are at least in part mediated by direct action on the α5‐GABAA receptors. Indeed, morphological investigations of spine density and dendritic length of adult neurons in old mice showed that chronic treatment reversed dendritic shrinkage. Interestingly, stopping the treatment for 1 week maintained increased spine density and dendritic length compared to untreated old mice, but the beneficial effects on cognitive function were lost, suggesting that efficacy of the treatment relies mostly on its direct activity on the α5‐GABAA‐R (Prevot et al. 2021). However, mood was not investigated in this study, and it remains a possibility that different behavioral domains require different effects of GL‐II‐73 to be present during treatment.

This study is not without limitations. The detailed mechanism of action responsible for this effect, downstream of the α5‐GABAA receptor potentiation, remains to be investigated. Another limitation is that only male mice were included, and further studies should be carried out to demonstrate similar efficacy in female mice. In addition, this study focused on cellular effects and did not assess the behavioral correlates of these effects. However, we previously showed that chronic GL‐II‐73 treatment (as in this study) had antidepressant potential and procognitive efficacy (Prevot et al. 2019). Finally, mice tested in this study were young adults, and it has been suggested that fluoxetine does not stimulate AHN during aging (Couillard‐Despres et al. 2009; McAvoy et al. 2015), whereas physical exercise does (Micheli et al. 2018). It would be of interest to investigate whether GL‐II‐73 increases AHN in older mice since reduced GABA levels are reported during normal and pathological aging (Cuypers et al. 2018), and GL‐II‐73 increases neuroplasticity in models of these conditions (Bernardo et al. 2025; Prevot et al. 2021).

## Disclosure

T.D.P., M.M., J.M.C., M.M., and E.S. are listed inventors on patents covering synthesis and use of GL‐II‐73. E.S. is Founder and Board Director of Damona Pharmaceuticals, a biopharma dedicated to bringing novel GABAergic compounds to the clinic for which the use of GL‐II‐73 is licensed in. D.J.D., I.M.D., J.P.G. and M.Y.M. declare no conflicts of interest.

## Supporting information


Data S1.

## Data Availability

The data that support the findings of this study are available from the corresponding author upon reasonable request.
