# Supplementary material for: Chronic α5‐GABA‐A Receptor Potentiation Promotes Mouse Adult Hippocampal Neurogenesis
Source: Hippocampus. 2025 Jun 13;35(4):e70019. doi: 10.1002/hipo.70019 (PMC12166276; doi:10.1002/hipo.70019)
Supplement: Supplementary file 1 — Data S1. [file HIPO-35-0-s001.docx]

Supplementary Material

**Chronic α5-GABA-A receptor potentiation promotes mouse adult hippocampal neurogenesis**

Thomas D. Prevot^1,2,3^, Michael Marcotte^1^, Denis J. David^4^, Indira Mendez-David^4^, Md Yeunus Mian^5^, James M. Cook^5^, Jean-Philippe Guilloux^4^ and Etienne Sibille^1,2,3*^

^1^Campbell Family Mental Health Research Institute of CAMH, 250 college street, Toronto, ON, M5T 1R8 Canada

^2^Department of Psychiatry, University of Toronto, 250 college street, Toronto, ON, M5T 1R8 Canada

^3^Department of Pharmacology and Toxicology, University of Toronto, Medical Sciences Building, 1 King's College Cir Room 4207, Toronto, ON, M5S 1A8, Canada

^4^Université Paris-Saclay, UVSQ, Centre de recherche en Epidémiologie et Santé des Populations (CESP), UMR 1018, CESP-Inserm, Team Moods, Faculté de Pharmacie, Bâtiment Henri MOISSAN, Orsay, France.

^5^ Department of Chemistry and Biochemistry, University of Wisconsin–Milwaukee, 3210 N Cramer Street, 53211, WI, USA

*Corresponding Authors: Etienne Sibille, Ph.D, CAMH, 250 College Street, room 134, Toronto, ON M5T 1R8, Canada. E-mail: Etienne.sibille@camh.ca

Running Title: Increase in α5-GABAA-R function stimulates adult hippocampal neurogenesis

Key Words: Adult hippocampal neurogenesis; GABA; preclinical; fluoxetine; hippocampus, α5-γ-Aminobutyric acid type A (GABAA) receptor

Disclosure: TP, MM, JMC, MM, and ES are listed inventors on patents covering synthesis and use of GL-II-73. ES is Founder and Chief Scientific Officer of Damona Pharmaceuticals, a biopharma dedicated to bringing novel GABAergic compounds to the clinic for which the use of GL-II-73 is licensed in. TP is the acting Director of Preclinical Research and Development in Damona. DD, ID, JPG and MYM declare no conflicts of interest.

**Materials and methods.**

***Ethical Statement***

Procedures involving animals and their care were conducted in accordance with the institutional guidelines and compliant with national and international laws and policies (Council directive # 87-848, October 19, 1987, Ministère de l'Agriculture et de la Forêt, Service Vétérinaire de la Santé et de la Protection Animale, permissions # 92-256B to DJD, Institutional Animal Care and Use Committee 26 authorization #4747).

***Animals***

Thirty (30) male 129S6/SvEvTac mice (Taconic Farms) (n= 10/group) were used in this study. Mice were 8-weeks-old at study initiation. The mice were maintained on a 12 L: 12 D schedule (lights on at 0700) and were housed in groups of five. Food and water were provided *ad libitum*. Treatments were administered one week after their arrival at the animal care facility. Animals were weighed weekly until the end of the experiment.

***Treatment***

The positive allosteric modulator at the α5-GABAA receptor, GL-II-73 (Batch # DK-IV-93-1), was dissolved in NaCl (0.9%) and given in mice during 6 weeks at a dose of 30mg/kg/day, p.o. (Bernardo, 2022; Prevot, 2020). The effects of GL-II-73 were compared to vehicle-treated mice and to fluoxetine-treated animals (18mg/kg/day, p.o, USBiological Ref#276420, Lot L18103154) (**Figure 1C** in main document). Each treatment group included 10 mice, however, one mouse from the fluoxetine treated group died early in the study.

To assess the survival level of newborn progenitor cells and whether fluoxetine or GL-II-73 treatment increases this process, 5-Bromo-2´-Deoxyuridine (BrdU; Roche Diagnostics, France, #10280879001) was dissolved in NaCl (0.9%) and injected 2x/day (100mg/kg, i.p.) for 3 days, 2 weeks before the end of the treatments.

***Perfusion and sectioning***

After completion of the treatment regimen (week 6), mice were anaesthetized with ketamine/xylazine (100 and 7 mg/kg, respectively; Ketamine: Merial, France; Xylazine: Bayer, France) and perfused transcardially (cold saline for 2 min, followed by 4% cold paraformaldehyde at 4°C for 1 min). Paraformaldehyde solution contains paraformaldehyde (4%), NaH_2_PO_4_ (0.1M) and Na_2_HPO_4_ (0.1M) (Sigma-Aldrich, France). The brains were then removed and cryoprotected in 30% sucrose (VWR, France) and stored at 4°C. Serial sections (35 μM) were cut through the entire hippocampus (plate 41-61 on (Franklin, 1997)) on a cryostat (Leica, Model CM3050s) and stored in PBS with 0.1% NaN3 (Sigma-Aldrich, France) before immunohistochemistry processing.

***Immunohistochemistry***

*Ki67 labeling for neural progenitor proliferation.* Sections were blocked in 0.3% triton in PBS and 10% NDS and incubated overnight at 4°C with anti-rabbit Ki67 (Ki67 rabbit, 1:100; Ref#VP-RM04, Eurobio, France). After washing with PBS, sections were incubated for 2 hr with secondary antibody (1:200 biotinylated donkey anti-rabbit, Jackson ImmunoResearch, Ref# #016-220-084).

*BrdU labeling for neuronal survival.* Sections were mounted on slides and boiled in citric acid (pH 6.0) for 5 min, rinsed with PBS, and treated with 0.01% trypsin in Tris/CaCl2 for 10 min. Brain sections were incubated for 30 min with 2N HCl and blocked with 5% Normal Goat Serum (NGS). Sections were then incubated overnight at room temperature with anti-mouse BrdU (BrdU; 1:100; Ref#347580, BD Biosciences, France). After washing with PBS, sections were incubated for 1 hr with secondary antibody (1:200 biotinylated goat anti-mouse, Ref #BA9200, Vector, France) followed by amplification with an avidin/biotin complex. The staining was visualized with 3,3'-diaminobenzidine (DAB). For the quantification of BrdU labelling, a stereological procedure was used as previously described (Malberg, 2000) with an Olympus BX51 (Germany) upright microscope.

*Doublecortin (DCX) labelling for neuronal maturation index.* For DCX staining, the procedure consisted of the following steps (David, 2009; Mendez-David, 2023). Sections were rinsed in TBS, treated with 1% H_2_O_2_ in TBS for 15 min to quench endogenous peroxidase activity (and to enhance dendritic staining), incubated in 10% normal donkey serum and 0.3% Triton X- 100 for 30 min, and then incubated overnight at 4°C in primary antibody for DCX (goat;1:500; Ref# #SC8066 Santa Cruz Biotechnology, Santa Cruz, CA). The secondary antibody was a biotinylated donkey anti-goat (1:500, Jackson ImmunoResearch, Ref #705-165-003, West Grove, PA) and incubated in TBS for 2 hr at room temperature. Sections were developed using avidin-biotin complex (Vector, USA,) and DAB kit (Vector, USA, SK-4105). DCX+ cells were subcategorized according to their dendritic morphology (Wang, 2008).

*Triple labelling for confocal analysis of neurogenesis.*  As DCX can be regulated independently of levels of neurogenesis (Mendez-David, 2023), we used triple labelling to test the fate of BrdU+ cells. Immunohistochemistry was performed using the following steps: 2 hr incubation in 1:1 formamide/2xSSC at 65°C, 5 min rinse in 2xSSC, 30 min incubation in 2N HCl at 37°C, and 10 min rinse in 0.1 M boric acid, pH 8.5, 2h incubation in 0.1 M PBS with 0.3% Triton X- 100, and 5% normal donkey serum. Sections were then incubated overnight at 4°C in primary antibodies for doublecortin (goat 1:500; Ref# #SC8066, Santa Cruz Biotechnology, Santa Cruz, CA), bromodeoxyuridine (BrdU; rat; 1:100; Ref#MCA2060, Serotec, Oxford, UK) and neuronal specific nuclear protein (NeuN) (mouse; 1:500; Ref#Mab377 Chemicon, Temecula, CA). Then fluorescent secondary antibodies were used. All secondary antibodies were purchased from Jackson ImmunoResearch (1:500 each: BrdU: CY2 conjugated Streptavidine, Ref# 016-220-084; DCX: Donkey anti-goat CY3, Ref#705-165-003; NeuN: Donkey anti mouse CY5, Ref# 715-605-150, Jackson ImmunoResearch, France) Approximately 6 sections per animal and 20-30 BrdU+ cells per treatment group were analyzed (n= 4-5 animals per conditions). Among the BrdU+ cells, the percentage of BrdU+/DCX+/NeuN-, BrdU+/DCX+/NeuN+ and BrdU+/DCX-/NeuN+ cells were evaluated for each treatment group.

***Imaging.***

All individual stainings (Ki67, BrdU and DCX) were visualized with DAB and quantified following a previously described method (Malberg, 2000) using an Olympus BX51 (Germany) upright microscope. For the triple labelling study, confocal imaging was performed on an Olympus IX83 P2ZF confocal microscope at 20X magnification. Triple Labelled cells were counted manually, applying a mask on ImageJ to count cells in the region of interest. Cells counts were normalized to the average area of the region of interest of areas measured.

***Statistical analysis.***

Data are presented as means ± S.E.M for Ki67, BrdU and DCX-positive cells. Normal distribution and presence of potential outliers was verified prior to statistical analysis. Dixon’s test identified one statistical outlier for BrdU, Ki67 and DCX staining and was removed from the analysis. Data were then analyzed by one-way ANOVA with treatment as main factor, followed by *post-hoc* analyses. Differences were considered significant at p≤0.05. All analysis were conducting using GraphPad Prism 6.

**Supplementary Data**

**Supplementary Figure 1. Chronic GL-II-73 treatment increases cellular proliferation and survival in dorsal and ventral HPC**


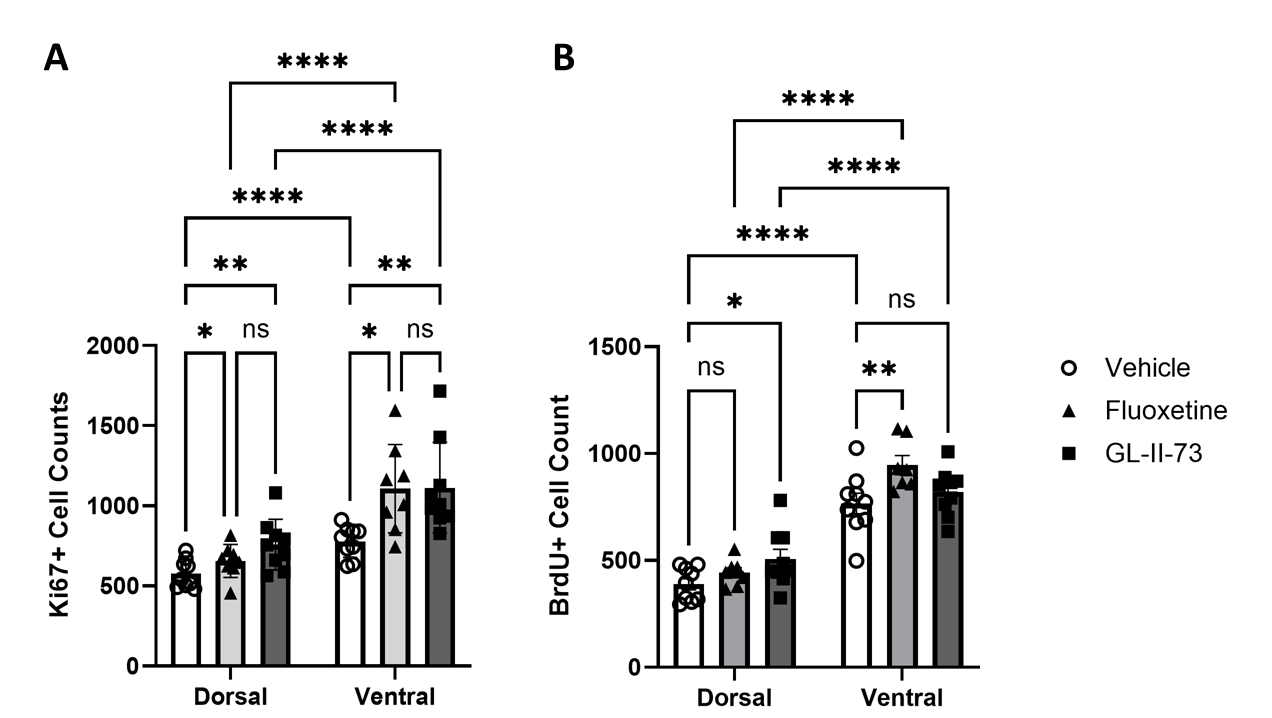


Regional analysis of proliferation and survival was conducted across the dorsal and ventral segments of the DG. Analysis of proliferation (**Supplementary Figure 1A**) by two-way ANOVA confirmed an effect of treatment (F_(2,23)_=10.67; p=0.0005) and region (F_(1,23)_=38.69; p<0.0001), though no significant interaction effect(F_(2, 23)_=1.828; p=0.18.34). *Post-hoc* analysis confirmed increase in Ki67 labelling with fluoxetine and GL-II-73 treatments compared to vehicle. Similar results were obtained in both the dorsal and the ventral segments, with *post-hoc* analyses showing higher Ki67 labelling in the ventral segment compared to the dorsal segment in both fluoxetine and GL-II-73 treated mice. Focusing on the BrdU labelling (**Supplementary Figure 1B**), 2-way ANOVA confirmed a significant effect of treatment (F_(2,22)_=3.991; p=0.0332), and showed a significant effect of region (F_(1,22)_=180.3; p=<0.0001) and a marginal interaction effect of treatment x region (F_(2,22)_=3.35; p=0.0537). GL-II-73 increased the number of BrdU+ Cells in the dorsal region (p=0.033) but not in the ventral region (p=0.326). Conversely fluoxetine increased proliferation in the ventral region (p=0.0031) but not in the dorsal region (p=0.355).

**Supplementary Figure 2. Chronic GL-II-73 increases neuronal maturation in the dorsal and ventral HPC**

Regional analysis of maturation index (**Supplementary Figure 2**), confirmed a significant effect of treatment (2-way ANOVA, F_(2,15)_=13.85; p=0.0004), and showed a significant effect of region (F_(1,15)_=7.73; p=0.0141) though no interaction treatment x region (F_(2,15)_=0.287; p=0.7545). *Post-hoc* analysis showed GL-II-73 increased the maturation index in the ventral region (p=0.0165), in the dorsal region the increase was marginal (p=0.059). Fluoxetine increased the maturation index in the ventral region (p=0.0002) and dorsal region (p=0.0004). Additionally for GL-II-73 treated mice maturation index was significantly higher in the ventral region (p=0.0143), though there were no regional differences for fluoxetine (p=0.342) or vehicle (p=0.1582) treated mice.


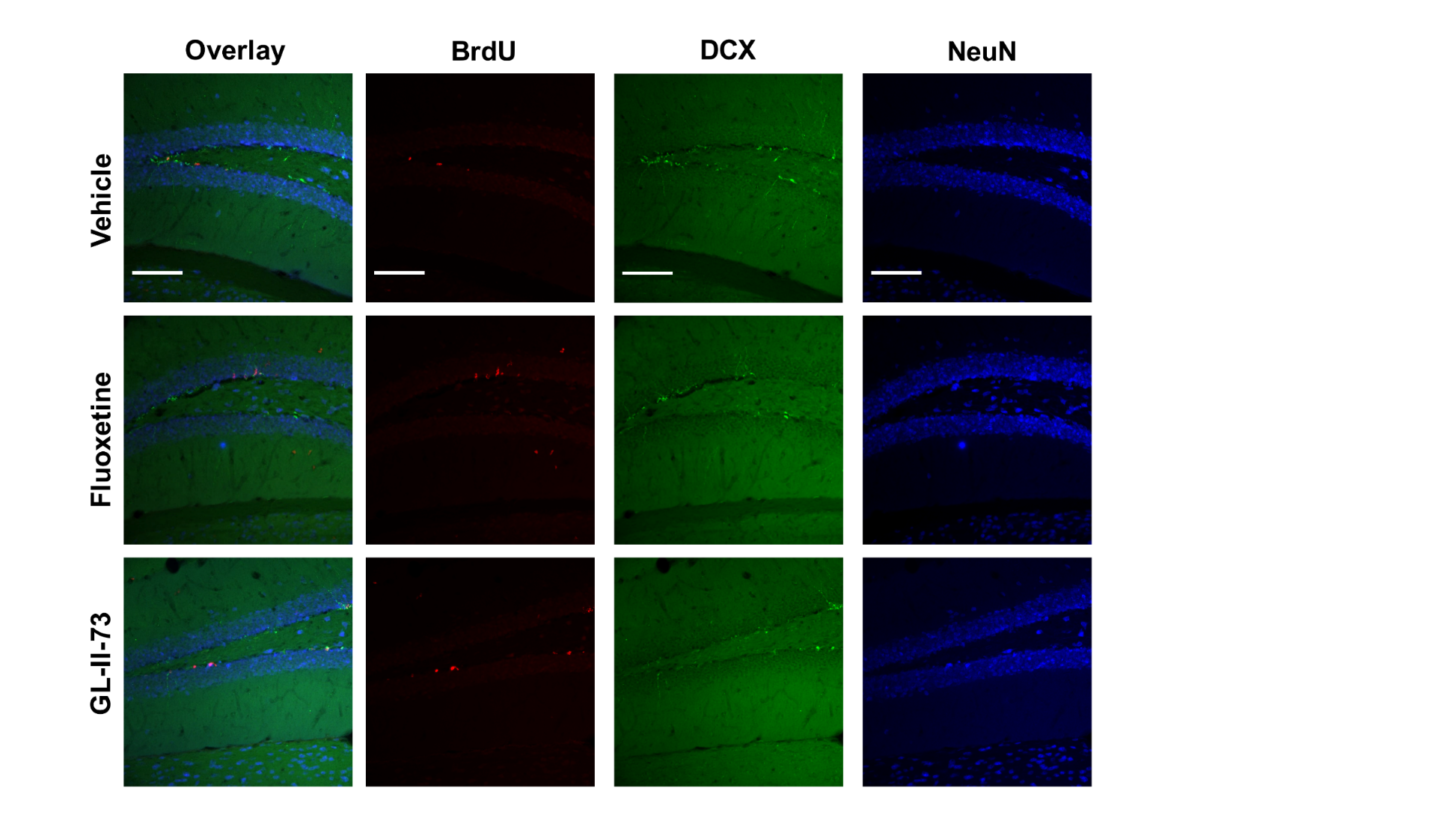
**Supplementary Figure 3. Representative images of triple labeling**

Hippocampal sections were stained with BrdU (Red), DCX (Green) and NeuN (NeuN). Cells were counted manually.

**Supplementary Figure 4. Effects of fluoxetine and GL-II-73 on changes in absolute and relative expression of DCX and NeuN in nascent neurons in the dorsal and ventral dentate gyrus**

**
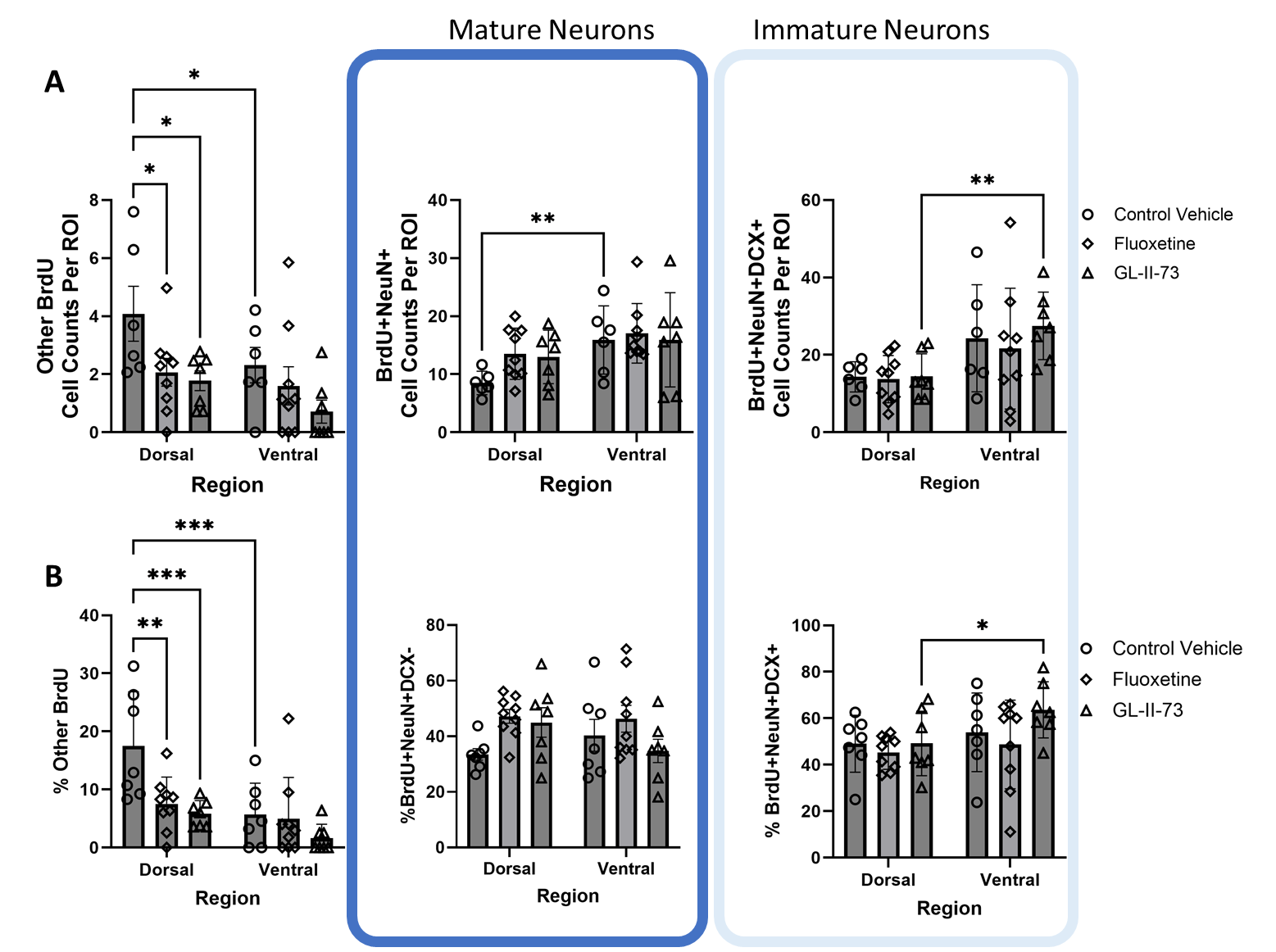
**

1. Analysis of cell type by region revealed significant of region (ANOVA F_(1, 19)_ = 6.145, p=0.0227) and treatment (F_(2,19)_=4.303, p = 0.0287) on other BrdU+ cells but no interaction effect. A significant effect of region was found for both BrdU+NeuN+ (ANOVA F_(2,19)_ =13.73, p=0.0015) and BrdU+NeuN+DCX+ (ANOVA F_(1,19)_=15.74, p=0.0008) but no effect of treatment. **B**) Analysis of relative expression of DCX and NeuN in BrdU positive cells also revealed a significant effect of region on other BrdU positive cells (ANOVA F_(1, 20)_ = 15.71, p=0.0008) and treatment (F_(2,20)_=5.717, p = 0.0109) though no interaction effect (ANOVA F_(2,20)_ = 3.292 p = 0.0581). For BrdU+NeuN+ cells no effect of region (ANOVA F_(1, 20)_ = 0.2383, p=0.6308) or treatment (ANOVA F_(2,20)_ = 2.195 p = 0.1374). For BrdU+DCX+NeuN+ cells there was a significant effect of region (ANOVA F_(1,20)_ = 5.216, p = 0.0334) but not treatment (ANOVA F_(2,20)_ = 1.266 p = 0.3037).

**Table of Statistical Analyses**

| Proliferation (Ki67) |  |  |  |  |  |
| --- | --- | --- | --- | --- | --- |
| Descriptive Statisitcs | VEH | FLX | GLII73 |  |  |
| N | 9 | 8 | 9 |  |  |
| Mean | 1352 | 1763 | 1847 |  |  |
| Std. Error of Mean | 36.11 | 91.18 | 92.53 |  |  |
|  |  |  |  |  |  |
| ANOVA summary |  |  |  |  |  |
| F | 12.21 |  |  |  |  |
| P value | 0.0002 |  |  |  |  |
| R squared | 0.515 |  |  |  |  |
| ANOVA table | **SS** | **DF** | **MS** | **F (DFn, DFd)** | **P value** |
| Treatment (between columns) | 1248580 | 2 | 624290 | F (2, 23) = 12.21 | P=0.0002 |
| Residual (within columns) | 1175870 | 23 | 51125 |  |  |
| Total | 2424450 | 25 |  |  |  |
| Post-Hoc Tests |  |  |  |  |  |
| Uncorrected Fisher's LSD | **Mean Diff.** | **95.00% CI of diff.** | **Below threshold?** | **Summary** | **Individual P Value** |
| VEH vs. FLX | -410.5 | -637.8 to -183.2 | Yes | ** | 0.0011 |
| VEH vs. GLII73 | -494.7 | -715.2 to -274.2 | Yes | *** | 0.0001 |
| FLX vs. GLII73 | -84.17 | -311.4 to 143.1 | No | ns | 0.4514 |

| Dorsal Ventral Proliferation | | | | | |
| --- | --- | --- | --- | --- | --- |
| ANOVA table | **SS** | **DF** | **MS** | **F (DFn, DFd)** | **P value** |
| Region x Treatment | 138116 | 2 | 69058 | F (2, 23) = 1.828 | P=0.1834 |
| Region | 1461681 | 1 | 1461681 | F (1, 23) = 38.69 | P<0.0001 |
| Treatment | 668565 | 2 | 334282 | F (2, 23) = 10.67 | P=0.0005 |
| Subject | 720415 | 23 | 31322 | F (23, 23) = 0.8290 | P=0.6717 |
| Residual | 869031 | 23 | 37784 |  |  |
|  |  |  |  |  |  |
|  |  |  |  |  |  |
| Uncorrected Fisher's LSD | **Predicted (LS) mean diff.** | **95.00% CI of diff.** | **Below threshold?** | **Summary** | **Individual P Value** |
| Dorsal |  |  |  |  |  |
| Vehicle vs. Fluoxetine | -79.5 | -261.3 to 102.3 | No | ns | 0.3833 |
| Vehicle vs. GL-II-73 | -181.3 | -357.7 to -4.949 | Yes | * | 0.0442 |
| Fluoxetine vs. GL-II-73 | -101.8 | -283.6 to 79.98 | No | ns | 0.2654 |
| Ventral |  |  |  |  |  |
| Vehicle vs. Fluoxetine | -331 | -512.8 to -149.2 | Yes | *** | 0.0006 |
| Vehicle vs. GL-II-73 | -337.3 | -513.7 to -160.9 | Yes | *** | 0.0004 |
| Fluoxetine vs. GL-II-73 | -6.333 | -188.1 to 175.5 | No | ns | 0.9444 |
| Vehicle |  |  |  |  |  |
| Dorsal vs. Ventral | -200 | -389.6 to -10.44 | Yes | * | 0.0395 |
| Fluoxetine |  |  |  |  |  |
| Dorsal vs. Ventral | -451.5 | -652.6 to -250.4 | Yes | *** | 0.0001 |
| GL-II-73 |  |  |  |  |  |
| Dorsal vs. Ventral | -356 | -545.6 to -166.4 | Yes | *** | 0.0007 |

| Survival (BrdU) |  |  |  |  |  |
| --- | --- | --- | --- | --- | --- |
| ANOVA summary |  |  |  |  |  |
| F | 3.991 |  |  |  |  |
| P value | 0.0332 |  |  |  |  |
| P value summary | * |  |  |  |  |
| Significant diff. among means (P < 0.05)? | Yes |  |  |  |  |
| R squared | 0.2662 |  |  |  |  |
| ANOVA table | **SS** | **DF** | **MS** | **F (DFn, DFd)** | **P value** |
| Treatment (between columns) | 241573 | 2 | 120786 | F (2, 22) = 3.991 | P=0.0332 |
| Residual (within columns) | 665843 | 22 | 30266 |  |  |
| Total | 907416 | 24 |  |  |  |
|  |  |  |  |  |  |
| Post-Hoc Tests |  |  |  |  |  |
| Uncorrected Fisher's LSD | Mean Diff. | 95.00% CI of diff. | Below threshold? | Summary | Individual P Value |
| VEH vs. FLX | -233 | -414.9 to -51.23 | Yes | * | 0.0144 |
| VEH vs. GLII73 | -171.3 | -341.4 to -1.254 | Yes | * | 0.0485 |
| FLX vs. GLII73 | 61.71 | -120.1 to 243.5 | No | ns | 0.4889 |

| Dorsal/Ventral Survival (BrdU) | | | | | |
| --- | --- | --- | --- | --- | --- |
| ANOVA table | **SS** | **DF** | **MS** | **F (DFn, DFd)** | **P value** |
| Region x Treatment | 72432 | 2 | 36216 | F (2, 22) = 3.350 | P=0.0537 |
| Region | 1949699 | 1 | 1949699 | F (1, 22) = 180.3 | P<0.0001 |
| Treatment | 120786 | 2 | 60393 | F (2, 22) = 3.991 | P=0.0332 |
| Subject | 332922 | 22 | 15133 | F (22, 22) = 1.400 | P=0.2184 |
| Residual | 237863 | 22 | 10812 |  |  |
| Uncorrected Fisher's LSD | **Predicted (LS) mean diff.** | **95.00% CI of diff.** | **Below threshold?** | **Summary** | **Individual P Value** |
| Dorsal |  |  |  |  |  |
| Vehicle vs. Fluoxetine | -53.62 | -169.3 to 62.06 | No | ns | 0.3553 |
| Vehicle vs. GL-II-73 | -118 | -226.2 to -9.792 | Yes | * | 0.0333 |
| Fluoxetine vs. GL-II-73 | -64.38 | -180.1 to 51.30 | No | ns | 0.2681 |
| Ventral |  |  |  |  |  |
| Vehicle vs. Fluoxetine | -179.4 | -295.1 to -63.75 | Yes | ** | 0.0031 |
| Vehicle vs. GL-II-73 | -53.33 | -161.5 to 54.87 | No | ns | 0.326 |
| Fluoxetine vs. GL-II-73 | 126.1 | 10.42 to 241.8 | Yes | * | 0.0333 |
| Vehicle |  |  |  |  |  |
| Dorsal vs. Ventral | -377.3 | -479.0 to -275.7 | Yes | **** | <0.0001 |
| Fluoxetine |  |  |  |  |  |
| Dorsal vs. Ventral | -503.1 | -618.4 to -387.9 | Yes | **** | <0.0001 |
| GL-II-73 |  |  |  |  |  |
| Dorsal vs. Ventral | -312.7 | -414.3 to -211.0 | Yes | **** | <0.0001 |

| Maturation (DCX) |  |  |  |  |  |
| --- | --- | --- | --- | --- | --- |
| ANOVA table | **SS (Type III)** | **DF** | **MS** | **F (DFn, DFd)** | **P value** |
| Interaction | 440065 | 2 | 220033 | F (2, 32) = 0.1489 | P=0.8622 |
| Tertiary | 50761890 | 1 | 50761890 | F (1, 32) = 34.36 | P<0.0001 |
| Treatment | 19284565 | 2 | 9642283 | F (2, 32) = 6.527 | P=0.0042 |
| Residual | 47272870 | 32 | 1477277 |  |  |
|  |  |  |  |  |  |
| Uncorrected Fisher's LSD | **Predicted (LS) mean diff.** | **95.00% CI of diff.** | **Below threshold?** | **Summary** | **Individual P Value** |
|  |  |  |  |  |  |
| Tertiary:VEH vs. Tertiary:FLX | -1577 | -3143 to -10.99 | Yes | * | 0.0485 |
| Tertiary:VEH vs. Tertiary:GLII73 | -355.7 | -1737 to 1025 | No | ns | 0.6034 |
| Tertiary:VEH vs. Total:VEH | -2280 | -3846 to -714.2 | Yes | ** | 0.0057 |
| Tertiary:VEH vs. Total:FLX | -4294 | -5859 to -2728 | Yes | **** | <0.0001 |
| Tertiary:VEH vs. Total:GLII73 | -2562 | -3943 to -1181 | Yes | *** | 0.0006 |
| Tertiary:FLX vs. Tertiary:GLII73 | 1221 | -159.8 to 2602 | No | ns | 0.0811 |
| Tertiary:FLX vs. Total:VEH | -703.2 | -2269 to 862.6 | No | ns | 0.3671 |
| Tertiary:FLX vs. Total:FLX | -2717 | -4283 to -1151 | Yes | ** | 0.0013 |
| Tertiary:FLX vs. Total:GLII73 | -985.6 | -2367 to 395.3 | No | ns | 0.1557 |
| Tertiary:GLII73 vs. Total:VEH | -1924 | -3305 to -543.4 | Yes | ** | 0.0078 |
| Tertiary:GLII73 vs. Total:FLX | -3938 | -5319 to -2557 | Yes | **** | <0.0001 |
| Tertiary:GLII73 vs. Total:GLII73 | -2207 | -3374 to -1040 | Yes | *** | 0.0005 |
| Total:VEH vs. Total:FLX | -2014 | -3579 to -447.8 | Yes | * | 0.0134 |
| Total:VEH vs. Total:GLII73 | -282.4 | -1663 to 1099 | No | ns | 0.6798 |
| Total:FLX vs. Total:GLII73 | 1731 | 350.3 to 3112 | Yes | * | 0.0156 |

| Maturation Index | | | | | |
| --- | --- | --- | --- | --- | --- |
| ANOVA table | **SS** | **DF** | **MS** | **F (DFn, DFd)** | **P value** |
| Treatment (between columns) | 0.08225 | 2 | 0.0411 | F (2, 16) = 14.12 | P=0.0003 |
| Residual (within columns) | 0.0466 | 16 | 0.0029 |  |  |
| Total | 0.1288 | 18 |  |  |  |
| Uncorrected Fisher's LSD | **Mean Diff.** | **95.00% CI of diff.** | **Below threshold?** | **Summary** | **Individual P Value** |
| VEH vs. FLX | -0.18 | -0.2524 to -0.1076 | Yes | **** | <0.0001 |
| VEH vs. GLII73 | -0.07378 | -0.1376 to -0.009968 | Yes | * | 0.0261 |
| FLX vs. GLII73 | 0.1062 | 0.04241 to 0.1700 | Yes | ** | 0.0028 |

| Dorsal/Ventral Maturation Index | | | | | |
| --- | --- | --- | --- | --- | --- |
| ANOVA table | **SS** | **DF** | **MS** | **F (DFn, DFd)** | **P value** |
| Dorso/ventral x Treatment | 0.001416 | 2 | 0.0007078 | F (2, 15) = 0.2871 | P=0.7545 |
| Dorso/ventral | 0.01899 | 1 | 0.01899 | F (1, 15) = 7.703 | P=0.0141 |
| Treatment | 0.1093 | 2 | 0.05467 | F (2, 15) = 13.85 | P=0.0004 |
| Subject | 0.05922 | 15 | 0.003948 | F (15, 15) = 1.601 | P=0.1860 |
| Residual | 0.03698 | 15 | 0.002466 |  |  |
| Uncorrected Fisher's LSD | **Predicted (LS) mean diff.** | **95.00% CI of diff.** | **Below threshold?** | **Summary** | **Individual P Value** |
| Dorsal |  |  |  |  |  |
| VEH vs. FLX | -0.1629 | -0.2405 to -0.08532 | Yes | *** | 0.0002 |
| VEH vs. GLII73 | -0.06201 | -0.1265 to 0.002499 | No | ns | 0.059 |
| FLX vs. GLII73 | 0.1009 | 0.03140 to 0.1704 | Yes | ** | 0.0059 |
| Ventral |  |  |  |  |  |
| VEH vs. FLX | -0.1508 | -0.2284 to -0.07319 | Yes | *** | 0.0004 |
| VEH vs. GLII73 | -0.08021 | -0.1447 to -0.01570 | Yes | * | 0.0165 |
| FLX vs. GLII73 | 0.07057 | 0.001069 to 0.1401 | Yes | * | 0.0468 |
| VEH |  |  |  |  |  |
| Dorsal vs. Ventral | -0.04664 | -0.1136 to 0.02029 | No | ns | 0.1582 |
| FLX |  |  |  |  |  |
| Dorsal vs. Ventral | -0.03451 | -0.1093 to 0.04033 | No | ns | 0.3412 |
| GLII73 |  |  |  |  |  |
| Dorsal vs. Ventral | -0.06484 | -0.1147 to -0.01495 | Yes | * | 0.0143 |

| Triple Labeling |  |  |  |  |  |
| --- | --- | --- | --- | --- | --- |
| ANOVA table | **SS** | **DF** | **MS** | **F (DFn, DFd)** | **P value** |
| Cell Type x Treatment | 367.8 | 4 | 91.96 | F (4, 40) = 1.276 | P=0.2957 |
| Cell Type | 9336 | 2 | 4668 | F (1.500, 30.01) = 64.75 | P<0.0001 |
| Treatment | 144 | 2 | 72 | F (2, 20) = 0.6362 | P=0.5397 |
| Subject | 2264 | 20 | 113.2 | F (20, 40) = 1.570 | P=0.1107 |
| Residual | 2884 | 40 | 72.09 |  |  |
| Uncorrected Fisher's LSD | **Mean Diff.** | **95.00% CI of diff.** | **Below threshold?** | **Summary** | **Individual P Value** |
| Other BrdU+ |  |  |  |  |  |
| VEH vs. FLX | 3.023 | 0.02583 to 6.019 | Yes | * | 0.0484 |
| VEH vs. GLII73 | 3.307 | 0.3952 to 6.219 | Yes | * | 0.0304 |
| FLX vs. GLII73 | 0.2844 | -1.599 to 2.168 | No | ns | 0.7506 |
| Brdu+NeuN+DCX- |  |  |  |  |  |
| VEH vs. FLX | -9.34 | -15.88 to -2.798 | Yes | ** | 0.0085 |
| VEH vs. GLII73 | -8.649 | -18.78 to 1.481 | No | ns | 0.0851 |
| FLX vs. GLII73 | 0.6912 | -9.696 to 11.08 | No | ns | 0.8851 |
| Brdu+NeuN+DCX+ |  |  |  |  |  |
| VEH vs. FLX | -3.142 | -19.98 to 13.70 | No | ns | 0.6935 |
| VEH vs. GLII73 | -4.026 | -19.14 to 11.08 | No | ns | 0.5638 |
| FLX vs. GLII73 | -0.8843 | -14.11 to 12.34 | No | ns | 0.8874 |
| VEH |  |  |  |  |  |
| Other BrdU+ vs. Brdu+NeuN+DCX- | -12.32 | -17.42 to -7.215 | Yes | ** | 0.001 |
| Other BrdU+ vs. Brdu+NeuN+DCX+ | -23.16 | -38.42 to -7.909 | Yes | ** | 0.0099 |
| Brdu+NeuN+DCX- vs. Brdu+NeuN+DCX+ | -10.85 | -22.37 to 0.6814 | No | ns | 0.0609 |
| FLX |  |  |  |  |  |
| Other BrdU+ vs. Brdu+NeuN+DCX- | -24.68 | -30.37 to -19.00 | Yes | **** | <0.0001 |
| Other BrdU+ vs. Brdu+NeuN+DCX+ | -29.33 | -41.61 to -17.05 | Yes | *** | 0.0006 |
| Brdu+NeuN+DCX- vs. Brdu+NeuN+DCX+ | -4.648 | -12.99 to 3.694 | No | ns | 0.2348 |
| GLII73 |  |  |  |  |  |
| Other BrdU+ vs. Brdu+NeuN+DCX- | -24.27 | -33.87 to -14.68 | Yes | *** | 0.0008 |
| Other BrdU+ vs. Brdu+NeuN+DCX+ | -30.5 | -37.62 to -23.37 | Yes | **** | <0.0001 |
| Brdu+NeuN+DCX- vs. Brdu+NeuN+DCX+ | -6.223 | -20.75 to 8.305 | No | ns | 0.3349 |

| % Triple Labeling |  |  |  |  |  |
| --- | --- | --- | --- | --- | --- |
| ANOVA table | **SS** | **DF** | **MS** | **F (DFn, DFd)** | **P value** |
| Cell Type x Treatment | 761.1 | 4 | 190.3 | F (4, 40) = 1.367 | P=0.2626 |
| Cell Type | 22893 | 2 | 11446 | F (1.253, 25.06) = 82.23 | P<0.0001 |
| Treatment | 1.213E-11 | 2 | 6.1E-12 | F (2, 20) = 1.262 | P=0.3046 |
| Subject | 9.607E-11 | 20 | 4.8E-12 | F (20, 40) = 3.451e-014 | P>0.9999 |
| Residual | 5568 | 40 | 139.2 |  |  |
| Uncorrected Fisher's LSD | **Mean Diff.** | **95.00% CI of diff.** | **Below threshold?** | **Summary** | **Individual P Value** |
| Other BrdU+ |  |  |  |  |  |
| VEH vs. FLX | 7.88 | -0.03492 to 15.79 | No | ns | 0.0508 |
| VEH vs. GLII73 | 9.067 | 1.302 to 16.83 | Yes | * | 0.0285 |
| Brdu+NeuN+DCX+ |  |  |  |  |  |
| VEH vs. FLX | 1.311 | -12.56 to 15.18 | No | ns | 0.8384 |
| VEH vs. GLII73 | -3.026 | -18.85 to 12.80 | No | ns | 0.6841 |
| Brdu+NeuN+DCX- |  |  |  |  |  |
| VEH vs. FLX | -9.191 | -17.58 to -0.7996 | Yes | * | 0.0341 |
| VEH vs. GLII73 | -6.04 | -19.30 to 7.223 | No | ns | 0.3318 |

| Triple Labeling Regional Other BrdU | |  |  | |  | |  | |  | |
| --- | --- | --- | --- | --- | --- | --- | --- | --- | --- | --- |
| ANOVA table | | **SS** | **DF** | | **MS** | | **F (DFn, DFd)** | | **P value** | |
| Region x Treatment | | 3.086 | 2 | | 1.543 | | F (2, 19) = 0.7304 | | P=0.4948 | |
| Region | | 12.98 | 1 | | 12.98 | | F (1, 19) = 6.145 | | P=0.0227 | |
| Treatment | | 25.68 | 2 | | 12.84 | | F (2, 19) = 4.303 | | P=0.0287 | |
| Subject | | 56.69 | 19 | | 2.984 | | F (19, 19) = 1.412 | | P=0.2294 | |
| Residual | | 40.14 | 19 | | 2.113 | |  | |  | |
| Uncorrected Fisher's LSD | | **Predicted (LS) mean diff.** | **95.00% CI of diff.** | | **Below threshold?** | | **Summary** | | **Individual P Value** | |
| Dorsal | |  |  | |  | |  | |  | |
| Control Vehicle vs. Fluoxetine | | 2.019 | 0.3162 to 3.723 | | Yes | | * | | 0.0214 | |
| Control Vehicle vs. GL-II-73 | | 2.307 | 0.5094 to 4.105 | | Yes | | * | | 0.0133 | |
| Fluoxetine vs. GL-II-73 | | 0.2879 | -1.341 to 1.916 | | No | | ns | | 0.7224 | |
| Ventral | |  |  | |  | |  | |  | |
| Control Vehicle vs. Fluoxetine | | 0.7127 | -0.9904 to 2.416 | | No | | ns | | 0.4022 | |
| Control Vehicle vs. GL-II-73 | | 1.597 | -0.2008 to 3.395 | | No | | ns | | 0.0801 | |
| Fluoxetine vs. GL-II-73 | | 0.8843 | -0.7442 to 2.513 | | No | | ns | | 0.2786 | |
| Control Vehicle | |  |  | |  | |  | |  | |
| Dorsal vs. Ventral | | 1.774 | 0.01748 to 3.530 | | Yes | | * | | 0.048 | |
| Fluoxetine | |  |  | |  | |  | |  | |
| Dorsal vs. Ventral | | 0.4672 | -0.9669 to 1.901 | | No | | ns | | 0.5035 | |
| GL-II-73 | |  |  | |  | |  | |  | |
| Dorsal vs. Ventral | | 1.064 | -0.5625 to 2.690 | | No | | ns | | 0.187 | |
| Triple Labeling Regional BrdU+ NeuN+ | | | | | | | | | |  |
| ANOVA table | **SS** | **DF** | | **MS** | | **F (DFn, DFd)** | | **P value** | |  |
| Region x Treatment | 37.98 | 2 | | 18.99 | | F (2, 19) = 1.139 | | P=0.3412 | |  |
| Region | 228.9 | 1 | | 228.9 | | F (1, 19) = 13.73 | | P=0.0015 | |  |
| Treatment | 70.33 | 2 | | 35.17 | | F (2, 19) = 0.8715 | | P=0.4344 | |  |
| Subject | 766.7 | 19 | | 40.35 | | F (19, 19) = 2.420 | | P=0.0306 | |  |
| Residual | 316.8 | 19 | | 16.68 | |  | |  | |  |
| Uncorrected Fisher's LSD | **Predicted (LS) mean diff.** | **95.00% CI of diff.** | | **Below threshold?** | | **Summary** | | **Individual P Value** | |  |
| Dorsal |  |  | |  | |  | |  | |  |
| Control Vehicle vs. Fluoxetine | -5.036 | -10.73 to 0.6613 | | No | | ns | | 0.0815 | |  |
| Control Vehicle vs. GL-II-73 | -4.49 | -10.50 to 1.524 | | No | | ns | | 0.139 | |  |
| Fluoxetine vs. GL-II-73 | 0.5461 | -4.902 to 5.994 | | No | | ns | | 0.8403 | |  |
| Ventral |  |  | |  | |  | |  | |  |
| Control Vehicle vs. Fluoxetine | -1.143 | -6.840 to 4.554 | | No | | ns | | 0.6869 | |  |
| Control Vehicle vs. GL-II-73 | -0.03715 | -6.051 to 5.977 | | No | | ns | | 0.9901 | |  |
| Fluoxetine vs. GL-II-73 | 1.106 | -4.342 to 6.554 | | No | | ns | | 0.6834 | |  |
| Control Vehicle | |  | |  | |  | |  | |  |
| Dorsal vs. Ventral | -7.408 | -12.34 to -2.473 | | Yes | | ** | | 0.0054 | |  |
| Fluoxetine | |  | |  | |  | |  | |  |
| Dorsal vs. Ventral | -3.515 | -7.544 to 0.5142 | | No | | ns | | 0.0836 | |  |
| GL-II-73 |  |  | |  | |  | |  | |  |
| Dorsal vs. Ventral | -2.955 | -7.524 to 1.614 | | No | | ns | | 0.1917 | |  |

| Triple Labeling Regional BrdU+ NeuN+ DCX+ | | | | | |
| --- | --- | --- | --- | --- | --- |
| ANOVA table | **SS** | **DF** | **MS** | **F (DFn, DFd)** | **P value** |
| Region x Treatment | 52.86 | 2 | 26.43 | F (2, 19) = 0.3660 | P=0.6983 |
| Region | 1137 | 1 | 1137 | F (1, 19) = 15.74 | P=0.0008 |
| Treatment | 84.64 | 2 | 42.32 | F (2, 19) = 0.3124 | P=0.7354 |
| Subject | 2574 | 19 | 135.5 | F (19, 19) = 1.876 | P=0.0897 |
| Residual | 1372 | 19 | 72.21 |  |  |
| Uncorrected Fisher's LSD | **Predicted (LS) mean diff.** | **95.00% CI of diff.** | **Below threshold?** | **Summary** | **Individual P Value** |
| Dorsal |  |  |  |  |  |
| Control Vehicle vs. Fluoxetine | 0.5834 | -10.29 to 11.46 | No | ns | 0.9141 |
| Control Vehicle vs. GL-II-73 | -0.09996 | -11.58 to 11.38 | No | ns | 0.986 |
| Fluoxetine vs. GL-II-73 | -0.6834 | -11.08 to 9.713 | No | ns | 0.8948 |
| Ventral |  |  |  |  |  |
| Control Vehicle vs. Fluoxetine | 2.659 | -8.214 to 13.53 | No | ns | 0.6234 |
| Control Vehicle vs. GL-II-73 | -3.202 | -14.68 to 8.275 | No | ns | 0.5755 |
| Fluoxetine vs. GL-II-73 | -5.861 | -16.26 to 4.535 | No | ns | 0.2609 |
| Control Vehicle |  |  |  |  |  |
| Dorsal vs. Ventral | -9.967 | -20.24 to 0.3022 | No | ns | 0.0564 |
| Fluoxetine |  |  |  |  |  |
| Dorsal vs. Ventral | -7.891 | -16.28 to 0.4934 | No | ns | 0.0636 |
| GL-II-73 |  |  |  |  |  |
| Dorsal vs. Ventral | -13.07 | -22.58 to -3.562 | Yes | ** | 0.0097 |

| Triple Labeling Regional BrdU+ NeuN+ DCX+ | | | | | |
| --- | --- | --- | --- | --- | --- |
| ANOVA table | **SS** | **DF** | **MS** | **F (DFn, DFd)** | **P value** |
| Region x Treatment | 52.86 | 2 | 26.43 | F (2, 19) = 0.3660 | P=0.6983 |
| Region | 1137 | 1 | 1137 | F (1, 19) = 15.74 | P=0.0008 |
| Treatment | 84.64 | 2 | 42.32 | F (2, 19) = 0.3124 | P=0.7354 |
| Subject | 2574 | 19 | 135.5 | F (19, 19) = 1.876 | P=0.0897 |
| Residual | 1372 | 19 | 72.21 |  |  |
| Uncorrected Fisher's LSD | **Predicted (LS) mean diff.** | **95.00% CI of diff.** | **Below threshold?** | **Summary** | **Individual P Value** |
| Dorsal |  |  |  |  |  |
| Control Vehicle vs. Fluoxetine | 0.5834 | -10.29 to 11.46 | No | ns | 0.9141 |
| Control Vehicle vs. GL-II-73 | -0.09996 | -11.58 to 11.38 | No | ns | 0.986 |
| Fluoxetine vs. GL-II-73 | -0.6834 | -11.08 to 9.713 | No | ns | 0.8948 |
| Ventral |  |  |  |  |  |
| Control Vehicle vs. Fluoxetine | 2.659 | -8.214 to 13.53 | No | ns | 0.6234 |
| Control Vehicle vs. GL-II-73 | -3.202 | -14.68 to 8.275 | No | ns | 0.5755 |
| Fluoxetine vs. GL-II-73 | -5.861 | -16.26 to 4.535 | No | ns | 0.2609 |
| Control Vehicle |  |  |  |  |  |
| Dorsal vs. Ventral | -9.967 | -20.24 to 0.3022 | No | ns | 0.0564 |
| Fluoxetine |  |  |  |  |  |
| Dorsal vs. Ventral | -7.891 | -16.28 to 0.4934 | No | ns | 0.0636 |
| GL-II-73 |  |  |  |  |  |
| Dorsal vs. Ventral | -13.07 | -22.58 to -3.562 | Yes | ** | 0.0097 |

| Triple Labeling Regional %BrdU+ NeuN+ | | | | | |
| --- | --- | --- | --- | --- | --- |
| ANOVA table | SS | DF | MS | F (DFn, DFd) | P value |
| Region x treatment | 514.6 | 2 | 257.3 | F (2, 20) = 2.805 | P=0.0844 |
| Region | 21.86 | 1 | 21.86 | F (1, 20) = 0.2383 | P=0.6308 |
| treatment | 826.4 | 2 | 413.2 | F (2, 20) = 2.195 | P=0.1374 |
| Subject | 3765 | 20 | 188.2 | F (20, 20) = 2.052 | P=0.0581 |
| Residual | 1835 | 20 | 91.74 |  |  |
| Uncorrected Fisher's LSD | Predicted (LS) mean diff. | 95.00% CI of diff. | Below threshold? | Summary | Individual P Value |
|  |  |  |  |  |  |
| Dorsal |  |  |  |  |  |
| Control Vehicle vs. Fluoxetine | -13.69 | -25.75 to -1.644 | Yes | * | 0.0269 |
| Control Vehicle vs. GL-II-73 | -11.51 | -24.29 to 1.276 | No | ns | 0.0764 |
|  |  |  |  |  |  |
| Ventral |  |  |  |  |  |
| Control Vehicle vs. Fluoxetine | -5.971 | -18.02 to 6.079 | No | ns | 0.3226 |
| Control Vehicle vs. GL-II-73 | 5.61 | -7.172 to 18.39 | No | ns | 0.3803 |
|  |  |  |  |  |  |
| Control Vehicle |  |  |  |  |  |
| Dorsal vs. Ventral | -6.891 | -17.57 to 3.788 | No | ns | 0.1933 |
|  |  |  |  |  |  |
| Fluoxetine |  |  |  |  |  |
| Dorsal vs. Ventral | 0.8321 | -8.586 to 10.25 | No | ns | 0.8556 |
|  |  |  |  |  |  |
| GL-II-73 |  |  |  |  |  |
| Dorsal vs. Ventral | 10.22 | -0.4551 to 20.90 | No | ns | 0.0596 |

| Triple Labeling Regional %BrdU+ NeuN+ DCX+ | | | | | |
| --- | --- | --- | --- | --- | --- |
| ANOVA table | SS | DF | MS | F (DFn, DFd) | P value |
| region x treatment | 265.6 | 2 | 132.8 | F (2, 20) = 1.064 | P=0.3637 |
| region | 650.8 | 1 | 650.8 | F (1, 20) = 5.216 | P=0.0334 |
| treatment | 695.6 | 2 | 347.8 | F (2, 20) = 1.266 | P=0.3037 |
| Subject | 5496 | 20 | 274.8 | F (20, 20) = 2.203 | P=0.0425 |
| Residual | 2495 | 20 | 124.8 |  |  |
|  |  |  |  |  |  |
|  |  |  |  |  |  |
| Uncorrected Fisher's LSD | Predicted (LS) mean diff. | 95.00% CI of diff. | Below threshold? | Summary | Individual P Value |
|  |  |  |  |  |  |
| Dorsal |  |  |  |  |  |
| Control Vehicle vs. Fluoxetine | 3.733 | -10.66 to 18.13 | No | ns | 0.6031 |
| Control Vehicle vs. GL-II-73 | -0.148 | -15.42 to 15.12 | No | ns | 0.9845 |
|  |  |  |  |  |  |
| Ventral |  |  |  |  |  |
| Control Vehicle vs. Fluoxetine | 5.241 | -9.156 to 19.64 | No | ns | 0.4662 |
| Control Vehicle vs. GL-II-73 | -9.656 | -24.93 to 5.614 | No | ns | 0.2086 |
|  |  |  |  |  |  |
| Control Vehicle |  |  |  |  |  |
| Dorsal vs. Ventral | -4.909 | -17.36 to 7.545 | No | ns | 0.4207 |
|  |  |  |  |  |  |
| Fluoxetine |  |  |  |  |  |
| Dorsal vs. Ventral | -3.401 | -14.38 to 7.582 | No | ns | 0.5257 |
|  |  |  |  |  |  |
| GL-II-73 |  |  |  |  |  |
| Dorsal vs. Ventral | -14.42 | -26.87 to -1.962 | Yes | * | 0.0255 |

**REFERENCES**

Bernardo, A., Lee, P., Marcotte, M., Mian, M. Y., Rezvanian, S., Sharmin, D., . . . Prevot, T. D. (2022). Symptomatic and neurotrophic effects of GABAA receptor positive allosteric modulation in a mouse model of chronic stress. *Neuropsychopharmacology, 47*(9), 1608-1619. doi:10.1038/s41386-022-01360-y

David, D. J., Samuels, B. A., Rainer, Q., Wang, J. W., Marsteller, D., Mendez, I., . . . Hen, R. (2009). Neurogenesis-dependent and -independent effects of fluoxetine in an animal model of anxiety/depression. *Neuron, 62*(4), 479-493. doi:10.1016/j.neuron.2009.04.017

Franklin, K., & Paxinos, G. (1997). A stereotaxic atlas of the mouse brain. *San Diego: Academic*.

Malberg, J. E., Eisch, A. J., Nestler, E. J., & Duman, R. S. (2000). Chronic antidepressant treatment increases neurogenesis in adult rat hippocampus. *J Neurosci, 20*(24), 9104-9110. doi:10.1523/JNEUROSCI.20-24-09104.2000

Mendez-David, I., David, D. J., Deloménie, C., Tritschler, L., Beaulieu, J. M., Colle, R., . . . Hen, R. (2023). A complex relation between levels of adult hippocampal neurogenesis and expression of the immature neuron marker doublecortin. *Hippocampus, 33*(10), 1075-1093. doi:10.1002/hipo.23568

Prevot, T. D., Sumitomo, A., Tomoda, T., Knutson, D. E., Li, G., Mondal, P., . . . Sibille, E. (2020). Reversal of Age-Related Neuronal Atrophy by α5-GABAA Receptor Positive Allosteric Modulation. *Cerebral Cortex, 31*(2), 1395-1408. doi:10.1093/cercor/bhaa310

Wang, J. W., David, D. J., Monckton, J. E., Battaglia, F., & Hen, R. (2008). Chronic fluoxetine stimulates maturation and synaptic plasticity of adult-born hippocampal granule cells. *J Neurosci, 28*(6), 1374-1384. doi:10.1523/JNEUROSCI.3632-07.2008
